# Local structural alignment of RNA with affine gap model

**DOI:** 10.1186/1753-6561-5-S2-S2

**Published:** 2011-05-28

**Authors:** Thomas King-Fung Wong, Brenda Wing-Yan Cheung, Tak-Wah Lam, Siu-Ming Yiu

**Affiliations:** 1 Department of Computer Science, The University of Hong Kong, Hong Kong

## Abstract

**Background:**

Predicting new non-coding RNAs (ncRNAs) of a family can be done by aligning the potential candidate with a member of the family with known sequence and secondary structure. Existing tools either only consider the sequence similarity or cannot handle local alignment with gaps.

**Results:**

In this paper, we consider the problem of finding the optimal local structural alignment between a query RNA sequence (with known secondary structure) and a target sequence (with unknown secondary structure) with the affine gap penalty model. We provide the algorithm to solve the problem.

**Conclusions:**

Based on an experiment, we show that there are ncRNA families in which considering local structural alignment with gap penalty model can identify real hits more effectively than using global alignment or local alignment without gap penalty model.

## Background

A non-coding RNA (ncRNA) is a RNA molecule that does not translate into proteins. It has been shown to be involved in many biological processes [1-4]. The number of ncRNAs within the human genome was underestimated before, but recently some databases reveal over 212,000 ncRNAs [5] and more than 1,300 ncRNA families [6]. Large discoveries of ncRNAs and their families show the possibilities that ncRNAs may be as diverse as protein molecules [7]. Identifying ncRNAs is an important problem in biological study. However, it is time consuming and there is no effective method to identify ncRNAs in a laboratory, predicting ncRNAs based on known ncRNAs using comparative computational approach is one of the promising directions to identify potential candidates for further verification.

Most of the computational approaches are based on the observation that if two different ncRNA molecules are in the same family (with similar biological functions), they usually exhibit similar sequences as well as secondary structures. One common approach [8-10] is as follows. We pick an ncRNA member of a family with known sequence and secondary structure (referred as the query), scan along a genomic sequence and for each possible region (referred as the target), perform an alignment between the query and the target to obtain a similarity measure to decide if the region is a potential ncRNA candidate for that family. The similarity measure may only base on the sequence or both the sequence and secondary structure (the latter case is referred as *structural alignment*). Along this direction, there are some approaches [11-14] that make use of secondary structure prediction tools to predict the secondary structure to be formed by the target assuming that it is an ncRNA before performing the alignment. The accuracy may, however, depend on the accuracy of the secondary structure prediction tools.

Instead of using one member of a family, some other approaches [15] use a set of ncRNAs from the same family to train a model (e.g. covariance model). Then, using this model to scan a genomic sequence to identify potential regions that are ncRNA candidates of that family. What information (sequence similarity and/or secondary structure) to be captured from the known ncRNAs depends on how we define the model. However, in some cases, we may not have enough known members in a family to train a model. In this paper, we focus on the problem that uses one known member as the query and align it with a target sequence. We remark that there are also other computational methods that identify ncRNAs without using known members in a family. For example, some try to identify ncRNAs by considering the stability of secondary structures formed by the substrings of a given genome [16]. This method may not be very effective because a random sequence with high GC composition also allows an energetically favorable secondary structure [17]. So, the comparative approach we described in the above is still one of the most popular approaches.

**Figure 1:**
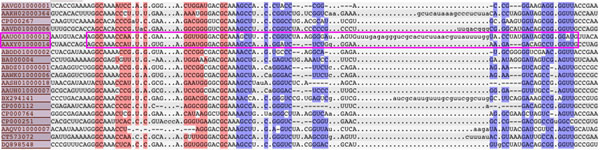
**Long gap may exist in conserved local region.** Multiple sequence alignment of some seed members of the family RF01051 from Rfam 9.1 database. The red and blue highlighted are the base-pair regions. All sequences are aligned according to their structures. If the two circled sequences are selected as query and target, the circled region is the conserved local region between them, in which there exists long gap inside.

The core idea behind all comparative approaches is to compute the similarity between the query (known member(s)) and the target (each possible region in the genomic sequence to be investigated). Some only consider sequence similarity which may not work well for families in which members do not have high sequence similarity (e.g. members of RF00017 in Rfam 9.1 [6] only have 39% sequence similarity). For example, Gotohscan [8] considers semi-global alignment with affine gap penalty according to the sequence similarity only. For those also consider the similarity of secondary structure, they usually require the whole sequence of the query to be aligned with the whole sequence of the target (referred as *global* alignment in the community) [10]. However, similar to the protein sequence, the ncRNAs in the same family may not have similar sequence or structure for the whole sequence but only for the substrings of them (those supposed to be the functional parts), especially when they belong to species with long evolutionary distance apart. Figure 1 shows one of these examples. It shows the multiple sequence alignment between some members of the family RF01051 in Rfam 9.1 database. The two circled members (i.e. AAUO01000012 and AAXYO1000014) are not quite similar if we consider the global alignment. Also, for the subregions that they look similar (i.e. the circled region), there exist large insertion/deletion (gaps). There are also evidences that gaps may be common in ncRNA homologs [18]. Considering local structural alignment with gap model seems to be more appropriate for predicting new members for some ncRNA families. [9] consider some restricted cases of local alignment according to the query structure. Another work that also consider local alignment is [11], but they cannot handle gaps.

We consider the following problem. Given a query sequence together with its secondary structure, we try to identify the substring in the given target sequence (with unknown secondary structure) that can align to a substring in the query sequence with the highest structural similarity score based on the affine gap model (see next section for formal definitions). We assume that the secondary structures of the ncRNAs are regular, that is, they do not have pseudoknots (no two base pairs crossing each other). This type of ncRNAs is found to be the most abundant in existing databases. We consider all possible substrings of the query sequence, even for those substrings that cover only one of the end points of some base pairs in the structure.

### Our result

We propose a local structural alignment algorithm with affine gap model which assumes the secondary structure of the query is known while that of the target sequence is unknown. The time complexity of our algorithm is *O*(*mn*^3^) which is the same as the best algorithm for global alignment for this problem where *m*, *n* are the lengths of the query and the target, respectively. We evaluated our algorithm using real data from Rfam database. According to the preliminary experiment, it shows that there are ncRNA families in which considering local structural alignment algorithm with affine gap model can distinguish real members from false hits more effectively than using global alignment or local alignment without affine gap model.

## Preliminaries

An ncRNA molecule can be regarded as a sequence of four characters {*A*, *C*, *G*, *U*}, each character is referred as a base. Some of these bases may form pairs (linked up by a hydrogen bond) with some restrictions such as each base can only pair up with at most one other base and only complementary bases can form a pair (e.g. (*A*, *U*), (*C*, *G*), (*U*,*G*)). The set of base pairs formed by the molecule is referred as its *secondary structure*.

Formally speaking, let *S* = *s*_1_*s*_2_ … *s_m_* be a length-*m* ncRNA sequence where *s_i_* ∈ {*A*, *C*, *G*, *U*} for 1 ≤ *i* ≤ *m* and *M* be the secondary structure of *S*. *M* is represented as a set of base pair positions. i.e. *M* = {(*i*, *j*)|1 ≤ *i* <*j* ≤ *m*, (*s_i_*, *s_j_*) is a base pair}. If (*s_i_*, *s_j_*) is base pair, then (*s_i_*, *s_j_*) ∈ {(*A*, *U*), (*C*, *G*), (*G*, *C*), (*G*, *U*), (*U*, *A*), (*U*, *G*)}. Let *M_x,y_* ⊆ *M* be the set of base pairs within the subsequence *s_x_s_x_*_+1_…*s_y_*, 1 ≤ *x* <*y* ≤ *m*, i.e., *M_x,y_* = {(*i*, *j*) ∈ *M|x* ≤ *i* <*j* ≤ *y*}. Note that if (*i*, *j*) ∈ *M* and only *i* or *j* inside the region [*x*…*y*], then (*i*, *j*) ∉ *M_x,y_*. We assume that there is no two base pairs sharing the same position, i.e., for any (*i*_1_, *j*_1_), (*i*_2_, *j*_2_) ∈ *M*, *i*_1_ ≠ *j*_2_, *i*_2_ ≠ *j*_1_, and *i*_1_ = *i*_2_ if and only if *j*_1_ = *j*_2_.

A regular structure is the structure in which there does not exist any two base pairs crossing each other. The formal definition is as follows:

**Definition 1***M_x,y_ is a regular structure if there does not exist two base pairs* (*i*, *j*), (*k*, *l*) ∈ *M_x,y_ such that i* <*k* <*j* <*lor k* <*i* <*l* <*j*.

Note that an empty set is also considered as a regular structure.

## Problem definition

### Structural alignment with affine gap model

Let *S*[1…*m*] be a query sequence with known secondary structure *M*, and *T*[1…*n*] be a target sequence with unknown secondary structure. *S* and *T* are both sequences of {A,C,G,U}. A structural alignment between *S* and *T* is a pair of sequences *S*′[1…*r*] and *T*′[1…*r*] where *r* ≥ *m*, *n*, *S*′ is obtained from *S* and *T*′ is obtained from *T* with spaces inserted to make both of the same length. A space cannot appear in the same position of *S*′ and *T*′. A maximal consecutive set of ℓ spaces in either *S*′ or *T*′ is referred as a gap of length ℓ. The score of the alignment (with affine gap penalty model), which determines the sequence and structural similarity between *S*′ and *T*′, is defined as *score* =

where *η*(*i*) is the corresponding position in *S* according to the position *i* in *S*′; *γ*(*u*_1_,*u*_2_) and *δ*(*u*_1_, *u*_2_, *v*_1_, *v*_2_) where *u*_1_, *u*_2_, *v*_1_, *v*_2_ ∈ {*A*, *C*, *G*, *U*}, are scores for character similarity and for base pair similarity respectively; *k* and *l* is the number of gaps and the total length of all gaps; *h* and *s* is the gap starting and extending penalty.

**Definition 2** An optimal global structural alignment *between S and T is a structural alignment of S and T such that the alignment score is maximum*.

Let *S*[*x*…*y*] where 1 ≤ *x*, *y* ≤ *m* be a substring of *S* with secondary structure *M_x,y_* (where *S*[*x*…*y*] is an empty string with empty structure if *x* >*y*). Similarly, let *T*[*x*′…*y*′] where 1 ≤ *x*′, *y*′ ≤ *n* be a substring of *T* (where *T*[*x*′…*y*′] is an empty string if *x*′ >*y*′).

**Definition 3** An optimal local structural alignment *between S and T is a global structural alignment between two sub stings of S and T, S*[*x*…*y*] *and T*[*x*′…*y*′] *where* 1 ≤ *x*, *y* ≤ *mand* 1 ≤ *x*′, *y*′ ≤ *n of S and T such that the alignment score between them is maximum over all possible substrings*.

Given *S* (with known secondary structure) and *T* (with unknown structure), we want to compute an optimal local structural alignment with affine gap penalty between *S* and *T*.

## Results and discussion

The details of the algorithm for solving the problem will be given in Method Section. In this section, we evaluate the resulting algorithm and show that considering local structural alignment with affine gap model can improve the effectiveness of locating ncRNAs for the families in which members may have variable size of hairpins, loops or stems when compared to using global alignment [10], local alignment without gap penalty model and Gotohscan [8]. Note that the differences in size of hairpins, loops or stems represent gaps in the corresponding sequences.

To test the algorithm, we selected around twenty ncRNA families in which the members have variable sizes of hairpins, loops or stems. We construct our testing cases based on real ncRNAs as follows. For each family, we first select a seed member (i.e. In Rfam database, there is a set of reliable members which are regarded as seed members) as the query sequence *Q*. To demonstrate the effectiveness of the affine gap model, we select the longest seed member as this query sequence. We then created a long random sequence with even distribution of four characters {*A*, *C*, *G*, *T*} to simulate a long genome. The length of this long random sequence is around ten times of the total length of all the seed members of the family. Finally, we embedded the full members (i.e. all the members including the seed members) of the family (except the one chosen as query) into this long random sequence in arbitrary positions. If there are more than 100 members, then we randomly picked 100 of them. This resulting sequence is our *T*.

Let *l* be the the maximum length of all the members of the family. For every region in *T* with length *l*+20, we compute the structural alignment score of the region and the query sequence. We use the same scoring scheme as in [9] and set the gap starting penalty (*h*) and gap extension penalty (*s*) to be 5 and 0.1, respectively. The details of the families including the sequence selected as the query, the length of the sequence, and the number of members embedded in each family are given in Table 1.

**Table 1:** The details of the ncRNA families used in the experiments.

Family	Query Sequence ID	Length	Number of members embedded
RF00014	CP000468.1/2032552-2032638	87	96
RF00021	CP000851.1/113395-113522	128	100
RF00022	AAND01000021.1/495-707	213	100
RF00027	AAPE01289140.1/8905-8994	90	100
RF00032	S49118.1/1081-1106	26	100
RF00033	Y15844.1/450-543	94	100
RF00034	BX571867.1/288515-288628	114	100
RF00038	AJ132964.1/66-198	133	100
RF00039	AF370716.1/3603-3656	54	100
RF00042	X55895.1/474-565	92	100
RF00043	Z47410.1/1220-1294	75	21
RF00044	M11813.1/4883-5126	244	8
RF00046	AY013245.2/62208-62303	96	76
RF00048	AF504534.1/666-726	61	100
RF00386	AF363455.1/1-122	122	100
RF00643	AASG02000279.1/67999-67862	138	100
RF00661	AC154049.1/4734-4855	122	100
RF01051	AE014299.1/1112481-1112574	94	100

We compare our algorithm with the global structural alignment [10], local structural alignment without affine gap model and Gotohscan [8]. Gotohscan was used to locate ncRNAs candidates on Trichoplax adhaerens by using single real ncRNA as query. It was designed to check only sequence similarity with affine gap model. Since the global structural alignment software is not available, we implemented both global and local without affine gap algorithms. For Gotohscan, we downloaded the version 1.3 from the website. We assume that regions other than the members of the family are false hits as they are likely not to be members of the family. To compute the effectiveness of our method, we use the smallest threshold with no false positive and the thresholds of allowing 5% or 10% false positive rate. We assume that the method finds a real hit if the score of the region is larger than this threshold. Thus a real hit will be missed if the computed score is smaller than or equal to this threshold. Table 2 and Table 3 summarize the result when using different algorithms to locate the other ncRNA members along the genome. When the smallest threshold with no false positive is used, the average percentage of misses when using Gotohscan is 53.9%; global alignment is 18.4%; local alignment without affine gap model is 17.9%; local alignment with affine gap model is 7.2%. When the threshold of allowing 5% or 10% false positive rate is used, the results show that the local structural alignment algorithm with affine gap model also works satisfactory except for the family RF00033. Table 4 also lists the area under the ROC curve when considering the false positive rate ≤ 10%. Note that the area is normalized to the range between 0 and 1.

**Table 2:** Summary of comparison on results between global alignment, local alignment without gap penalty and local alignment with affine gap penalty when using the smallest threshold such that there is no false positive.

Family	Number of members	Number of misses										
		
		Gotohscan [8]	%		Global [10]	%		Local	%		Local with affine gap	%
RF00014	96	2	2.1%		0	0%		0	0%		0	0%
RF00021	100	10	10%		5	5%		5	5%		2	2%
RF00022	100	59	59%		20	20%		19	19%		4	4%
RF00027	100	100	100%		15	15%		9	9%		2	2%
RF00032	100	59	59%		4	4%		1	1%		0	0%
RF00033	100	29	29%		27	27%		27	27%		25	25%
RF00034	100	71	71%		11	11%		22	22%		7	7%
RF00038	100	88	88%		0	0%		0	0%		0	0%
RF00039	100	100	100%		1	1%		1	1%		1	1%
RF00042	100	10	10%		0	0%		0	0%		0	0%
RF00043	21	3	14.3%		0	0%		0	0%		0	0%
RF00044	8	1	12.5%		0	0%		0	0%		0	0%
RF00046	76	9	11.8%		2	2.6%		1	1.3%		0	0%
RF00048	100	17	17%		0	0%		0	0%		0	0%
RF00386	100	88	88%		63	63%		62	62%		6	6%
RF00643	100	98	98%		4	4%		13	13%		0	0%
RF00661	100	100	100%		87	87%		77	77%		30	30%
RF01051	100	100	100%		91	91%		85	85%		52	52%
		**average**	**53.9%**			**18.4%**			**17.9%**			**7.2%**

**Table 3:** Summary of comparison on results between global alignment, local alignment without gap penalty and local alignment with affine gap penalty when setting the threshold which allows 5% or 10% of false positives.

Family	Number of members	Number of misses								
		
		False positive rate=5%					False positive rate=10%			
		
		Gotohscan	Global	Local	Local with affine gap		Gotohscan	Global	Local	Local with affine gap
RF00014	96	2	0	0	0		2	0	0	0
RF00021	100	10	1	1	1		10	1	1	1
RF00022	100	51	9	5	2		35	4	4	2
RF00027	100	100	3	5	0		100	2	2	0
RF00032	100	59	0	0	0		37	0	0	0
RF00033	100	27	1	25	24		26	1	1	24
RF00034	100	71	1	0	0		71	1	0	0
RF00038	100	88	0	0	0		88	0	0	0
RF00039	100	100	0	0	0		100	0	0	0
RF00042	100	10	0	0	0		10	0	0	0
RF00043	21	3	0	0	0		3	0	0	0
RF00044	8	1	0	0	0		1	0	0	0
RF00046	76	9	0	0	0		9	0	0	0
RF00048	100	11	0	0	0		11	0	0	0
RF00386	100	88	58	56	1		88	48	38	1
RF00643	100	98	1	4	0		98	0	2	0
RF00661	100	100	87	66	23		100	81	52	14
RF01051	100	100	79	85	47		100	79	81	39

**Table 4:** Summary of the area (normalized) under ROC curve for false positive rate ≤ 10%

Family	Area (normalized) under ROC curve			
	
	Gotohscan	Global	Local	Local with affine gap
RF00014	0.98	1.0	1.0	1.0
RF00021	0.9	0.99	0.99	0.99
RF00022	0.53	0.92	0.93	0.98
RF00027	0.0	0.96	0.96	1.0
RF00032	0.61	0.99	1.0	1.0
RF00033	0.73	0.93	0.79	0.76
RF00034	0.29	0.98	0.99	0.99
RF00038	0.12	1.0	1.0	1.0
RF00039	0.0	1.0	1.0	1.0
RF00042	0.9	1.0	1.0	1.0
RF00043	0.86	1.0	1.0	1.0
RF00044	0.88	1.0	1.0	1.0
RF00046	0.88	1.0	1.0	1.0
RF00048	0.89	1.0	1.0	1.0
RF00386	0.12	0.42	0.49	0.98
RF00643	0.02	0.99	0.96	1.0
RF00661	0.0	0.14	0.36	0.79
RF01051	0.0	0.18	0.17	0.56

**Figure 2:**
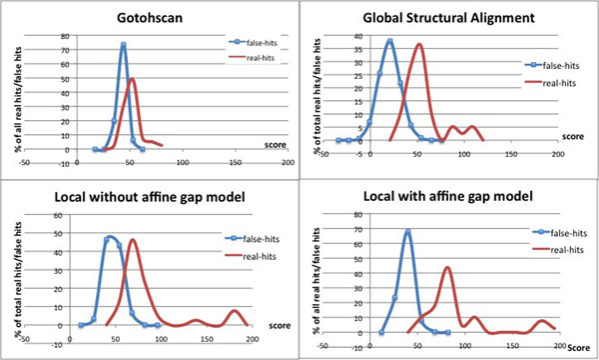
**Score distribution between the real hits and the false hits when using different algorithms for the family RF00661.** The figure shows the comparison on score distribution of real hits (i.e. real members) and false hits for the family RF00661 between different algorithms. It shows that the local structural alignment algorithm with affine gap penalty can increase the difference between the scores of real hits and the scores of false hits compared with the other methods, and so it has a higher distinguishing power to identify the real ncRNA members along the long genome sequence.

We also use RF00661 as an example and show the score distribution between the real hits and the false hits when using different algorithms in Figure 2. As one can see, the local structural alignment algorithm with affine gap penalty can increase the difference between the scores of real hits and the scores of false hits compared with the other methods, and so it has a higher distinguishing power to identify the real ncRNA members along the long genome sequence for these families.

Our program take around 15 seconds for performing local structural alignment with affine gap model between query and target of around 150 bases long, and around 30 seconds for 200 bases long. We tested the program on a machine with 2.4GHz dual-core CPU and 8G memory.

## Conclusions

In the paper, we provided an algorithm to handle local structural alignment with affine gap model of RNA with regular structure that compute the optimal alignment. Our experiments show that the solution is effective for some ncRNA families in which members may have varying sizes on hairpins, loops or stems (contributing to large gaps) when compared to using only global alignment or local alignment without gap model. And also we have not yet studied different types of gap penalty model and the effect of setting different gap penalty parameters. Other interesting directions include speeding up the algorithm and considering other more complicated structures (e.g. the structures with pseudoknots). In the mean time, we have completed the algorithm of computing local structural alignment for simple pseudoknots structure, and we are now in the progress of performing experiments.

## Methods

We develop a dynamic programming algorithm to solve the problem. Before we describe the method, we would like to define some variations of alignments which will be used in our algorithm. Let *S*[1…*m*] be the query sequence with known structure *M* and *T*[1…*n*] be the target sequence with unknown structure.

**Definition 4** Optimal prefix-global structural alignment *between S*[1…*m*] *and T*[1…*n*] *is to find a prefix S*[1…*y*] *where* 0 ≤ *y* ≤ *m(i.e. S is an empty string when y* = 0*) such that the score of the optimal global structural alignment between the prefix S*[1…*y*] *and T*[1…*n*] *is maximum*.

**Definition 5** Optimal suffix-global structural alignment *between S*[1…*m*] *and T*[1…*n*] *is to find S*[*x*…*m*] *where* 1 ≤ *x* ≤ *m* + 1 (*i.e. S is an empty string when x* = *m* + 1) *such that the score of the optimal global structural alignment between the suffix S*[*x*…*m*] *and T*[1…*n*] *is maximum*.

**Definition 6** Optimal semi-global structural alignment *between S*[1…*m*] *and T*[1…*n*] *is to find a substring S*[*x*…*y*] *where* 1 ≤ *x*, *y* ≤ *m such that the score of the optimal global structural alignment between the substring S*[*x*…*y*] *and T*[1…*n*] *is maximum*.

Let the affine gap model be *h* + *sL*, where *h* is the gap opening penalty, *s* represents a gap extension penalty, and *L* denotes the length of gap. Our method consists of two steps. In the first step, we compute the optimal semi-global structural alignment between *S* and all possible substrings of *T*. In the second step, we obtain the optimal local structural alignment between *S* and *T* resulted in the first step. Define *A*(*p*, *q*, *e*, *f*) to be the score of the optimal *semi-global* structural alignment between *S*[*p*…*q*] and *T* [*e*…*f*]. The score of the optimal *local* structural alignment between *S* and *T* can be obtained from the entry max*_e_*_≤_*_f_*_+1_*A*(1, *m*, *e*, *f*). We first show how to compute *A*, then show how to use the structure of *S* to guide the computation of *A* without considering all possible combinations of *p*, *q*.

When considering any substring *S*′ = *S*[*x*′…*y*′] of *S*[*x*…*y*], there are four possible cases: (1) *S*′ is equal to *S* (i.e. *x*′ = *x*, *y*′ = *y*); (2) *S*′ is a proper prefix in *S* (i.e. *x*′ = *x*, *y*′ <*y*); (3) *S*′ is a proper suffix in *S* (i.e. *x*′ >*x*, *y*′ = *y*); (4) *S*′ is a substring of *S*[*x* + 1…*y* – 1] (i.e. *x*′ >*x*, *y*′ <*y*); Therefore, we can consider each case one by one when computing the value of *A*.

Define *A*_1_(*p*, *q*, *e*, *f*) to be the score of the optimal *global* structural alignment between *S*[*p*…*q*] and *T*[*e*…*f*]. Define *A*_2_(*p*, *q*, *e*, *f*) to be the score of the optimal *prefix-global* structural alignment between *S*[*p*…*q* – 1] and *T*[*e*…*f*]. Define *A*_3_(*p*, *q*, *e*, *f*) to be the score of the optimal *suffix-global* structural alignment between *S*[*p* + 1…*q*] and *T*[*e*…*f*]. Define *A*_4_(*p*, *q*, *e*, *f*) to be the score of the optimal *semi-global* structural alignment between *S*[*p* + 1…*q* – 1] and *T*[*e*…*f*].

The value of *A*(*p*, *q*, *e*, *f*) can be computed recursively and it is the maximum value of four cases: (1) when *S*′ = *S*[*p*, *q*] (i.e. *A*_1_(*p*, *q*, *e*, *f*)); (2) when *S*′ is a proper prefix of *S*[*p*, *q*] (i.e. *A*_2_(*p*, *q*, *e*, *f*)); (3) when *S*′ is a proper suffix of *S*[*p*, *q*] (i.e. *A*_3_(*p*, *q*, *e*, *f*); (4) when *S*′ is a substring of *S*[*p* + 1, *q* – 1] (i.e. *A*_4_(*p*, *q*, *e*, *f*); Lemma 1 summarizes these cases.

Lemma 1

The following subsections describe how to compute *A*_1_,*A*_2_,*A*_3_,*A*_4_.

### Calculation of *A*_1_

When considering the optimal global structural alignment (with affine gap model) between *S*[*p*…*q*] and *T* [*e*…*f*], there are nine possible cases: (1) *S*[*p*] is aligned with *T*[*e*] and *S*[*q*] with *T*[*f*]; (2) *S*[*p*] with *T*[*e*] and *S*[*q*] with space;(3) *S*[*p*] with *T*[*e*] and *T*[*f*]*withspace*; (4) *S*[*p*] with space and *S*[*q*] with *T*[*f*]; (5) *S*[*p*] with space and *S*[*q*] with space; (6) *S*[*p*] with space and *T*[*f*] with space; (7) *T*[*e*] with space and *S*[*q*] with *T*[*f*]; (8) *T*[*e*] with space and *S*[*q*] with space; (9) *T*[*e*] with space and *T*[*f*] with space. Hence, we can consider each case one by one when computing the value of *A*_1_.

Define *A*_1_*_x_*(*p*, *q*, *e*, *f*), where 1 ≤ *x* ≤ 9, to be the score of the optimal *global* structural alignment between *S*[*p…q*] and *T*[*e…f*] where the above case *x* is satisfied. (i.e. if *x* = 1, then *S*[*p*] is aligned with *T*[*e*] and *S*[*q*] with *T*[*f*]).

The value of *A*_1_(*p*, *q*, *e*, *f*) can be computed recursively and it is the maximum value of nine cases. Lemma 2 summarizes these cases.

Lemma 2

We will describe the calculation of *A*_12_. Similar skill can be applied for the others (i.e. *A*_11_, *A*_13_, … , *A*_19_).

#### Calculation of A_12_

*A*_12_(*p*, *q*, *e*, *f*) is the score of the optimal global structural alignment between *S*[*p…q*] and *T*[*e…f*], which aligns *S*[*p*] with *T*[*e*] and *S*[*q*] with space. There are three situations and we need to consider them one by one. Note that according to the affine gap model, the penalty of a first space in a gap (i.e. which is *h* + *s*) is different from the penalty of the other space in a gap (i.e. which is *s*). Situation I: when (*p*, *q*) is a base pair - aligning the base pair *S*[*p*] with *T*[*e*] and *S*[*q*] with space. Considering the alignment between *S*[*p* + 1…*q* – 1] and *T*[*e* + 1…*f*], if *S*[*q* – 1] is aligned with space (i.e. case 2, case 5 and case 8), then a penalty *s* should be considered. Otherwise (i.e. for the other six cases), a penalty *h* + *s* should be considered. Situation II: when ∃*q*′ where *p* <*q*′ <*q* such that (*p*, *q*′) is a base pair - we need to find *k* ∈ [*e* – 1, *f*] such that the sum of the alignment score between *S*[*p*, *q*′] and *T*[*e*, *k*], and that between *S*[*q*′ + 1, *q*] and *T*[*k* + 1, *f*] is maximum. Since *S*[*p*] is aligned with *T*[*e*] and *S*[*q*] with space, the alignment between *S*[*p*,*q*′] and *T*[*e*, *k*] should satisfy the case 1, case 2 and case 3 (i.e. *S*[*p*] is aligned with *T*[*e*]). Similarly, the alignment between *S*[*q*′ + 1, *q*] and *T*[*k* + 1, *f*] should satisfy the case 2, case 5 and case 8 (i.e. *S*[*q*] is aligned with space). Situation III: when *p* does not form base pair with any base *q*′ ∈ [*p*, *q*] - we align base *S*[*p*] with *T*[*e*]. Then the alignment between *S*[*p* + 1…*q*] and *T*[*e*+ 1…*f*] should satisfy the case 2, case 5 and case 8 (i.e. *S*[*q*] is aligned with space). Lemma 3 summarizes these situations:

Lemma 3

### Calculation of *A*_2_

When considering the optimal prefix-global structural alignment (with affine gap model) between *S*[*p*…*q*] and *T*[*e*…*f*], there are four possible cases: (1) *S*[*p*] is aligned with *T*[*e*]; (2) *S*[*p*] with space; (3) *T*[*f*] with space; and (4) an empty string of *S* with *T*.

Define *A*_2*x*_(*p*, *q*, *e*, *f*), where 1 ≤ *x* ≤ 3, to be the score of the optimal *prefix-global* structural alignment between *S*[*p*…*q*] and *T*[*e*…*f*] where the above case *x* is satisfied. (i.e. if *x* = 1, then *S*[*p*] is aligned with *T*[*e*]). Note that we do not need to define function for the case 4 because the corresponding score is – *h – s*(*f – e* + 1). The value of *A*_2_(*p*, *q*, *e*, *f*) can be computed recursively and it is the maximum value of four cases. Lemma 4 summarizes these cases.

Lemma 4

*A*_2_(*p*, *q*, *e*, *f*) = max{*A*_21_[ *p*, *q*, *e*, *f*], *A*_22_[*p*, *q*, *e*, *f*], *A*_23_[*p*, *q*, *e*, *f*], – *h* – *s*(*f* – *e* + 1)}

We will describe the calculation of *A*_22_. Similar skill can be applied to calculate *A*_21_ and *A*_23_.

#### Calculation of A_22_

The following lemma lists out the computation of *A*_22_.

Lemma 5

*A*_22_(*p*, *q*, *e*, *f*) is the score of the optimal prefix-global structural alignment between *S*[*p*…*q* – 1] and *T*[*e*…*f*], where *S*[*p*] is aligned with space. Similar to *A*_12_, there are also the same three situations. Situation I: when (*p*, *q*) is a base pair - aligning the base pair *S*[*p*] with space. Since a prefix of *S*[*p*…*q* – 1] is considered, there are two possibilities: a prefix of *S*[*p* + 1…*q* – 1] is aligned with *T*[*e*…*f*] (i.e. semi-global alignment), or the whole sequence *S*[*p*+ 1…*q* – 1] is aligned with *T*[*e*…*f*] (i.e. global alignment). Situation II: when ∃*q*′ where *p* <*q*′ <*q* such that (*p*, *q*′) is a base pair - we need to find *k* ∈ [*e* – 1, *f*] such that the sum of the alignment score between *S*[*p*, *q*′] and *T*[*e*, *k*], and that between *S*[*q*′ + 1, *q*] and *T*[*k* + 1, *f*] is maximum. Since a prefix of *S*[*p*…*q* – 1] is considered, there are two possibilities: (1) the whole sequence *S*[*p*, *q*′] is aligned with *T*[*e*, *k*] (i.e. global alignment) and a prefix of *S*[*q*′ + 1, *q*] is aligned with *T*[*k* + 1, *f*] (i.e. semi-global); (2) a prefix of *S*[*p*, *q*′] is aligned with *T*[*e*, *k*] (i.e. semi-global) only. Situation III: when *p* does not form base pair with any base *q*′ ∈ [*p*, *q*] - we align base *S*[*p*] with space. For each possibility of situation I & III, there are also two conditions: if *S*[*p* + 1] is aligned with *T*[*e*] or *T*[*e*] is aligned with space, the penalty score *h* + *s* should be considered. Otherwise, if *S*[*p* + 1] is aligned with space, then the penalty score *s* should be considered. The lemma 5 summarizes these cases.

The calculations for *A*_3_ and *A*_4_ are similar. In the following subsection, we will describe the time complexity of the algorithm.

### Time complexity

To fill the dynamic programming table, not all entries for all possible subrange of *S* needs to be filled. According to the design of the dynamic programming, there are three cases:

Case 1: if (*p*, *q*) ∈ *M_p,q_*, then all the entries for *S*[*p*, *q*] of all tables (i.e. *A*, *A*_1_, *A*_2_, *A*_3_, *A*_4_, *A*_11_, …, etc.) can be computed from the entries for *S*[*p* – 1, *q* + 1].

Case 2: if ∃*q*′ <*q* s.t. (*p*, *q*′) ∈ *M_p,q_*, then all the entries for *S*[*p*, *q*] of all tables can be computed from the entries for *S*[*p*, *q*′] and *S*[*q*′ + 1, *q*].

Case 3: if ∄*q*′ s.t. (*p*, *q*′) ∈ *M_p,q_*, then all the entries for *S*[*p*, *q*] of all tables can be computed from the entries for *S*[*p* + 1, *q*].

Therefore, we define a function *ζ*(*p*, *q*) to determine for which set of subregions in *S*, we need to fill the corresponding entires in all the tables.

We only need to fill in the entries for all the tables provided (*p*, *q*) can be obtained from (1, *m*) by applying *ζ* function repeatedly. Considering the *ζ* function, each time the total size of the subregions outputted cannot be greater than the size of the input region and each of the subregions outputted is smaller than the input region. Therefore, in total there are only *O*(*m*) such (*p*, *q*) values. Also, there are *O*(*n*^2^) values of different (*e*, *f*) values, and for each entry, it takes *O*(*n*) because of the consideration of *e* – 1 ≤ *k* ≤ *f* in the case that ∃*q*′ <*q* s.t. (*p*, *q*′) ∈ *M_p,q_*. After finishing the calculation of values *A*(1, *m*, *e*, *f*) for all 1 ≤ *e*, *f* ≤ *n*, the final answer (i.e. max_*e*≤*f*+1_{*A*(1, *m*, *e*, *f*)}) can be computed in *O*(*n*^2^) time. Therefore the total time complexity = *O*(*mn*^3^) + *O*(*n*^2^) = *O*(*mn*^3^).

**Theorem 1***For any sequence S*[1..*m*] *with regular structure and any sequence T*[1…*n*] *with unknown structure, the optimal local alignment score between S*[1*..m*] *and T*[1..*n*] *can be computed in O*(*mn*^3^).

## Competing interests

The authors declare that they have no competing interests.

## Authors' contributions

TW and SY conceived the study. All authors refined the study. TW and BC came up the algorithm and BC implemented it. All authors contributed to the analysis. TW, SY and TL participated in drafting the manuscript. All authors read and approved the final version.
